# Pain Alleviation and Opioid Weaning in an 80-Year-Old with Chronic Foot Pain Following Injection Therapy with Perineural Dexmedetomidine and Dexamethasone

**DOI:** 10.1055/s-0040-1722176

**Published:** 2021-01-07

**Authors:** Amnon A. Berger, Ivan Urits, Jamal Hasoon, Alan D. Kaye, Omar Viswanath, Jonathan Eskander

**Affiliations:** 1 Department of Anesthesia, Critical Care, and Pain Medicine, Beth Israel Deaconess Medical Center, Harvard Medical School, Boston, Massachusetts; 2 Department of Anesthesiology, Louisiana State University Health Shreveport, Shreveport, Louisiana; 3 Valley Anesthesiology and Pain Consultants – Envision Physician Services, Phoenix, Arizona; 4 Department of Anesthesiology, University of Arizona College of Medicine - Phoenix, Phoenix, Arizona; 5 Department of Anesthesiology, Creighton University School of Medicine, Omaha, Nebraska; 6 Anesthesiology and Pain Medicine, Portsmouth Anesthesia Associates, Portsmouth, Virginia

**Keywords:** pain therapy, chronic pain, opioid withdrawal, Dex-Dex

## Abstract

Opiates are routinely used for chronic pain patients, and up to 44% of them will have a prescription for an opiate medication for pain alleviation. However, of the 76 million adults prescribed opiates for pain management, about 12% report misuse, and a large number of these may find themselves addicted to opioid medications. Opioid addiction is an ongoing epidemic, costing many lives. Withdrawal is very difficult. This requires providers to consider alternative analgesic plans and minimize opiate use. Here we report the use of a dexamethasone-dexmedetomidine combination for a regional nerve block in an elderly woman chronically treated with opiate medications who had previously failed opiate weaning. Following her nerve block, she was able to completely wean off of opioids and continues having good pain control with an opioid-free regimen.

Morphine is a potent analgesic and is often treated as the opiate prototype. Up to 44% of chronic pain patients are prescribed an opiate for pain alleviation. 1 Through reward pathway activation, morphine treats pain, but there is also a risk of opioid addiction. 2 It is estimated that of the 76 million adults were prescribed opioid drugs in 2016 to 2017, and 12% reported opioid misuse, often the first step leading to addiction. 3 In 2017, United States recorded 47,506 opioid deaths (13.21 per 100,000, up from 2.20 in 2004). 4 Withdrawal is often difficult, and relapse rates are high.

We briefly report the case of an 80-year-old patient who was previously diagnosed with chronic foot pain and treated with opiate medication for longer than a decade. She has had numerous previous foot and ankle surgeries, including arthroscopy, neuroma excision, and Achilles tendon lengthening; however, she continued to have chronic foot pain, mostly affecting the medial and dorsal aspects of her foot, which she described as a constant burning sensation with intermitted superimposed sharp pain. The pain was neuropathic in nature and 7/10 in severity on a 10-point scale, with exacerbation up to 10/10 with touch and activity. She was managed with increased doses of opiates, and she has been taking 80 mg of morphine equivalents for the 10 years preceding her presentation. Previous attempts of weaning down opioid doses failed due to the persistence of pain.

Most recently, this patient enrolled in a detoxification program targeting opioid detoxification within 1 to 2 weeks. This program uses a combination of weak opioids, benzodiazepines, promethazine, and possibly buprenorphine-naloxone (Saboxone), during outpatient treatment. Given the previous failure of detoxification and refractory pain, the patient underwent injection therapy to address her pain. Previous cases demonstrate prolonged nerve blockade using the dexmedetomidine and dexamethasone (Dex-Dex) combo. 5 6 7 She was given an adductor canal block, using a mixture of 0.2% ropivacaine with 5 mg preservative-free dexamethasone and 25 mcg of dexmedetomidine, alongside a popliteal block with local anesthetic and dexamethasone, to achieve full coverage. We decided to not use the Dex-Dex combo for the popliteal fossa block, given its ability to produce an unpredictably long sensory blockade along the common peroneal distribution.

Following this procedure, the patient has had complete pain relief for approximately 1 week and was able to wean off of opioid use entirely, from which she continues to abstain. Her overall pain was minimal at baseline and up to 1 to 2/10, with exacerbation during this 1 week follow-up. Her pain is now successfully managed using a combination of medical marijuana and cannabidiol (CBD) oil, which she uses about once weekly.

The actual mechanism of the Dex-Dex combination is unknown. Dexmedetomidine is a α2 receptor agonist. which is known to provide analgesia through supraspinal, spinal, and peripheral actions. Local action is known to prolong the effect of local anesthetics, and the mechanism is most probably via hyperpolarization-activated cation currents. Dexamethasone likely prolongs the effect of local anesthetics through its anti-inflammatory properties, most probably through the inhibition of prostaglandin formation and release of endorphins. Previous data found a synergistic effect of the combination, with a yet-to-be-discovered mechanism or action. 5 6 7

This case demonstrates an example of including injection therapy as an adjunct for opioid detoxification to address chronic pain in long-term opioid users. By addressing the pain component in this patient, we were able to potentiate medical therapy addressing the other aspects of withdrawal and detoxification and allow for a safe and continued opioid rehabilitation. This multimodal approach was successful in achieving long-term detoxification in a chronic opioid user. With the growing opioid misuse epidemic, there is a need for continually evolving approaches to facilitate detoxification and injection therapy is an important adjunct.
